# RNA Interference and Its Key Targets for Spinal Cord Injury Therapy: What Is Known So Far?

**DOI:** 10.3390/ijms26209861

**Published:** 2025-10-10

**Authors:** Daria Chudakova, Vladimir Kovalev, Matthew Shkap, Elizaveta Sigal, Arthur Biktimirov, Alesya Soboleva, Vladimir Baklaushev

**Affiliations:** 1 Federal Center for Brain and Neurotechnologies, Federal Medical and Biological Agency of Russia, 117513 Moscow, Russia; 2 Engelhardt Institute of Molecular Biology, Russian Academy of Sciences, 119991 Moscow, Russia; 3 Federal Scientific and Clinical Center for Specialized Types of Medical Care and Medical Technologies, Federal Medical and Biological Agency of Russia, 115682 Moscow, Russia; 4 Department of Medical Nanobiotechnology, Medical and Biological Faculty, Pirogov Russian National Research Medical University, Ministry of Health of the Russian Federation, 117997 Moscow, Russia

**Keywords:** spinal cord injury, SCI, RNA interference, RNAi, mesenchymal stromal cells, MSCs, translational research

## Abstract

Spinal cord injury (SCI) is a neurological condition often resulting in permanent motor and sensory deficits, for which effective treatments remain limited. RNA interference (RNAi) is a post-transcriptional mechanism of the downregulation of gene expression mediated by small interfering RNAs. RNAi has demonstrated therapeutic efficacy in various neurological disorders, positioning it as a promising yet underexplored therapeutic strategy for SCI. Here, we provide a focused overview of the key pathological processes in SCI, including primary mechanical injury and secondary cascades such as inflammation, mitochondrial dysfunction, excitotoxicity, oxidative stress, multiple forms of cell death, and others. The potential of RNAi to selectively silence genes implicated in these pathological processes, thereby enhancing neuroprotection and functional recovery, is highlighted. We point out that not only protein-coding genes, but non-coding RNAs (ncRNAs) are suitable targets for RNAi. Novel RNAi tools such as CRISPR-Cas13 might revolutionize the field and offer new opportunities for SCI therapy. However, despite all these promising findings, relevant translational studies of RNAi remain scarce. Challenges related to delivery methods, long-term efficacy, and cell-specific targeting must be addressed. Importantly, combining RNAi with other strategies such as cell- or biomaterial-based therapies may enhance therapeutic outcomes. Future investigations should prioritize systematic comparisons of RNAi targets and delivery systems, ideally at single-cell resolution and in different SCI models, to identify the most relevant molecular pathways for clinical translation. Overall, RNAi represents a compelling but still underdeveloped approach for SCI therapy, requiring continued refinement to reach clinical application.

## 1. Introduction

**Figure 1:**
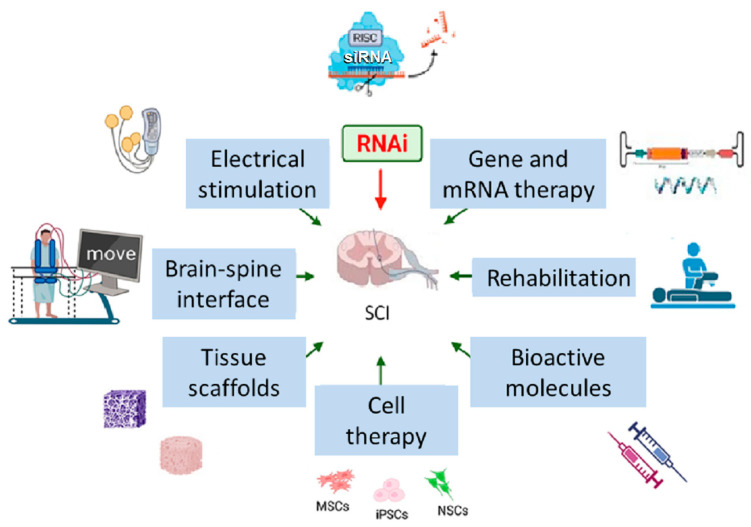
Current and emerging therapies for SCI. MSCs—mesenchymal stromal cells, iPSCs—induced pluripotent stem cells, NSCs—neural stem cells. The figure was designed and created using Biorender (accessed on 22 June 2025), Server Medical Art (licensed under a Creative Common Attribution 3.0 Unported License; https://smart.servier.com (accessed on 2 March 2024) and Bioicons (licensed under a Creative Common license; https://bioicons.com (accessed on 2 March 2024).

Spinal cord (SC) injury (SCI) is a debilitating condition caused by spinal trauma or, in some cases, by non-traumatic causes. It can be either complete or incomplete depending on the extent of preserved sensory and motor function below the site of injury. The annual incidence of SCI worldwide is estimated at around 15–40 cases per million [1]. Severe SCI leads to permanent functional impairment and affected individuals often endure incurable neurological dysfunctions including motor and sensory deficits, autonomic dysfunction, and chronic pain, all of which profoundly diminish their quality of life and impose significant financial burden on the healthcare system [2]. The central nervous system (CNS) of adult humans has very limited regeneration capacity; therefore, functions of an injured SC cannot be completely restored through neuroregeneration. Current treatment strategies for SCI primarily focus on surgical decompression, rehabilitation, physical therapy and symptomatic management; they are inadequate in addressing the underlying neurodegenerative processes within the injured spinal cord. Thus, there is an urgent need for novel therapies. One such promising curative approach is RNA interference (RNAi) therapy (Figure 1), a technology which mimics the RNAi naturally occurring in cells—a process in which short non-coding so-called interfering RNAs decrease the expression of specific genes by targeting their mRNAs for degradation or preventing their translation.

RNAi therapy commonly utilizes short hairpin RNAs (shRNAs), single-stranded antisense RNA oligonucleotides (ASO) and double-stranded small interfering RNAs (siRNAs) [3]. The molecular mechanisms of RNAi are comprehensively described elsewhere, including in the context of nerve injury treatment [4]. As a natural mechanism of gene expression regulation occurring in all cells under both normal and pathological conditions, RNAi plays a vital role in development and differentiation, as well as CNS-specific processes such as neurogenesis, myelination, axonal growth, etc. RNAi is particularly promising in modulating the complex and multifaceted pathological mechanisms of SCI, as RNAi-based drugs can target either one target precisely, which is common for synthetic small interfering RNAs, or multiple targets with partial complementarity, which is common for RNAi based on endogenous microRNAs. For example, MicroRNA miR-199b modulates the course of SCI by targeting multiple targets, in the NF-κB signaling pathway and in the RGMA/STAT3 [5,6], with all targets contributing to the pathophysiology of SCI. The possibility to modulate the expression of multiple genes in the injured SC by the siRNA “cocktails” (combination of several siRNA) is also being explored [7]. Importantly, RNAi can target not only coding, but also non-coding regulatory RNAs, which broadens its potential application [8]. It can also be used in the case of targets which are “undruggable” via small molecules, as well as targets whose mode of action cannot be modulated by antibodies. Overall, although challenges (not discussed in this review in detail, but hindering development of RNAi-based therapies) such as poor stability of RNAi molecules, their low delivery efficiency in vivo, immunogenicity, different uptake of RNAi molecules by different types of cells, the risk of off-target effects, and potential neurotoxicity of the viral vectors used for delivery of RNAi tools remain, continued advancements and optimization of RNAi technologies hold promise for their application in SCI treatment.

The therapeutic potential of RNAi is further evidenced by the fact that six siRNA-based drugs have been already approved for clinical use (Patisiran, approved for primary hyperoxaluria; Givosiran, for hereditary transthyretin-mediated amyloidosis (hATTR) with polyneuropathy and for acute hepatic porphyria; Inclisiran for hypercholesterolemia; Lumasiran for primary hyperoxaluria type 1; Vutrisiran for hATTR with polyneuropathy; and Nedosiran for primary hyperoxaluria).

In this narrative review, we summarize recent or seminal works on previously characterized RNAi targets for the treatment of SCI and propose several novel ones yet to be tested. Other subjects related to RNAi in the context of SCI, such as advanced delivery systems, off-target effects, timing and dosing, etc., are left beyond the scope of this concise review. We also focus on studies using RNAi with one known target rather than studies utilizing miRNAs with multiple targets.

The literature search and selection were performed as follows: Search in PubMed, Google Scholar and other open source databases was performed using keywords “SCI”, “spinal cord injury”, “RNAi”, “knockdown”, “siRNA”, “shRNA”, “RNA interference” in different combinations, aiming to select either recent articles from 2020 to 2025 or pioneering/seminal ones from earlier years. From more than 300 articles found using this approach, and several relevant related articles found via Google Scholar, the “core” articles to be reviewed were selected by two co-first authors of this manuscript, based on their significance, novelty, and relevance to the scope of this review.

## 2. SCI: Sequence of Events

SCI involves complex molecular and cellular events that begin immediately following trauma. It can be divided into primary and secondary injuries, with the latter sub-divided into acute, sub-acute, and chronic phases, comprehensively described in numerous works elsewhere [9] and concisely described below. In the context of SCI, RNAi can be used to modulate many steps of its pathological processes.

The primary injury—an immediate phase, damage of the SC—results in the damage of cells within the SC (including rupture of long axons) and adjacent blood vessels. SCI also causes significant disruption and remodeling of the extracellular matrix’s (ECM) architecture and, hence, its function. All of this initiates a cascade of molecular events leading to secondary injuries. Secondary injury might be divided into acute (first 48 h post-injury), sub-acute (from 48 h to about 14 days post-injury) and chronic phases, although several different classifications of SCI stages exist [10,11]. The pathological processes during the acute phase include mitochondrial dysfunction and cellular energy production catastrophe, ion imbalance and excitotoxicity, inflammation, edema, hypoxia, and others, all of which result in mostly necrotic cell death. This is followed by the sub-acute phase, characterized by mostly apoptotic cell death, massive demyelination, axonal “die-back” (withdrawal of the tips of axons proximal to the lesion), Wallerian degeneration (anterograde degeneration in the distal segment of injured axons), further ECM remodeling, and formation of astroglial scars. Finally, the chronic phase features cystic cavity formation (syringomyelia), ongoing axonal “die-back”, and fibrotic scar development.

## 3. Targets for RNAi in SCI

### 3.1. ECM Remodeling and Scar Formation

In the SC, the ECM forms a complex, three-dimensional scaffold that supports cell migration and axonal pathfinding. The ECM adjacent to neurons forms so-called Perineuronal Nets (PNNs)—highly condensed form of the ECM; damage of PNNs as a result of SCI might lead to decreased resistance of neurons to neurotoxic stimuli [12]. Physical properties of the ECM, such as stiffness, porosity, and viscoelasticity, are recognized by growing axons and hence modulate their guidance and motility. Axonal pathfinding is also guided by the interaction of transmembrane receptors (such as integrins) with ECM proteins (such as fibronectin and laminin), and with so-called guidance molecules (semaphorins, slits, ephrins, etc.), which can be found on cell surfaces or within ECM. The post-SCI, altered ECM regulates local inflammation, gliosis, cell survival and differentiation (including the fate of neural stem cells) [13], ultimately affecting endogenous regeneration [14]. For example, the early post-SCI ECM negatively affects neural stem cells’ viability, impairing their regenerative potential [13]. The ECM’s impact on inflammation is mostly executed via so-called damage-associated molecular patterns (DAMPs) (endogenous molecules released by damaged cells that trigger innate immune response, also known as alarmins) and by immunomodulatory chondroitin sulfate proteoglycans (CSPGs), such as aggrecan, brevican, neurocan, versican, phosphacan, etc. [15]. Alarmins promote inflammation through Toll-like receptors (TLRs), receptors for advanced glycation end products (RAGE), and G protein-coupled receptors (GiPCRs) [16], whereas CSPGs can prevent inflammation resolution and block phenotypic conversion of immune cells in SCI [17] but are primarily known to inhibit axonal growth [18]. Overall, it has been suggested that the complexity of alarmin signaling—single alarmins engaging multiple receptors and pathways—poses challenges for designing effective RNAi interventions [16]. Nevertheless, several works focus on the RNAi of alarmins signaling in SCI. It has been shown that RNAi which decreases the levels of enzyme **chondroitin polymerizing factor (ChPF)** involved in the synthesis of CSPG glycosaminoglycan results in a less inhibitory environment for axonal growth [19]. It is known that enzymatic degradation of CSPGs by chondroitinase ABC (ChABC) promotes axonal growth and functional recovery. However, ChABC’s effect lasts in vivo only for 1–2 days, whereas RNAi of ChPF can sustain reduced CSPG levels for more than week, thus offering a longer therapeutic window for axon regeneration [19]. Notably, immediate inhibition of CSPG synthesis post-injury impairs motor recovery and increases tissue loss, whereas inhibition starting 2 days post-injury enhances axonal regeneration and functional recovery [15]. An example of the dual role of some molecules in post-SCI recovery is Tenascin-C. Tenascin-C is a multifunctional ECM protein whose levels are elevated in response to SCI. Given its involvement in the regulation of inflammation and tissue remodeling [20] it receives attention as an RNAi therapy target in CNS trauma [21]. However, it is also capable of promoting axonal growth in some contexts, for example, when, as a therapeutic approach, the expression of specific Tenascin-binding integrins (such as α9β1) is activated in neurons in the presence of integrin activator kindlin-1 [22].

Furthermore, damage of myelin caused by SCI leads to the exposure and release of several myelin-associated inhibitors (MAI) of axonal growth/sprouting, such as neurite outgrowth inhibitor A (Nogo-A), oligodendrocyte myelin glycoprotein (OMgp), and myelin-associated glycoprotein (MAG). **RNAi approaches targeting the Nogo pathway** have been explored in the context of SCI in several publications (summarized in [23] with encouraging results). It should be added that Nogo-A can also be targeted by antibodies, and the corresponding results obtained in human studies of SCI are promising [24]. Notably, the expression of Nogo-A dynamically varies post-SCI; it decreases within 24 h post-injury, reaches a low at 3 days, peaks around 7 days and remains elevated at 14 days, illustrating the importance of precise timing of RNAi targeting Nogo-A [25].

Myelin debris generated by SCI persists for extended periods, contributing to the chronic inhibition of axonal growth and remyelination. Thus, its timely removal by phagocytic cells is crucial. Within the ECM, levels of repulsive guidance molecule A (RGMa) are significantly elevated after SCI. This molecule is expressed by neurons, oligodendrocytes, and astrocytes, presents in both the glial scar and myelin debris at the SC injury site, and acts as a neurite growth inhibitor. Thus, RGMa or its receptor Neogenin are considered promising therapeutic targets for SCI, as evidenced by preclinical studies and animal models using approaches other than RNAi [26]. However, there are no examples of RNAi targeting RGMa specifically in SCI yet. Notably, one of the pathways activated by inhibitory molecules like RGMa and MAI, contributing to growth cone collapse, is the **RhoA/Rho-associated kinase (ROCK) pathway**, one of the well-known therapeutic targets in SCI (discussed in detail further in the text).

Within the ECM, so-called axon guidance molecules (semaphorins, ephrins, etc.) become dysregulated post-SCI and can block normal axonal pathfinding and impair the “re-building” of neural circuits essential for functional recovery. Data obtained in animal models (rats) demonstrate that the RNAi of **EphrinB3** improves functional recovery following SCI [27]. Furthermore, the delivery of siRNA targeting **semaphorin 3A (sem3A)** increased cell survival, neuronal differentiation and synaptic connectivity of the transplanted embryonal neural stem cells (NSCs) post-SCI in rats [28].

After trauma of the SC, astrocytes become polarized and generally acquire an A1 (neurotoxic) or A2 (neuroprotective) phenotype [29]; they mainly respond to SCI by forming a dense scar of so-called reactive astrocytes (hypertrophic astrocytes secreting pro-inflammatory mediators) around the injury site, isolating damaged tissues and preventing further spread of injury. An astrocytic scar, while protective, becomes a barrier impeding axonal regeneration. As for the ECM, reactive astrocytes secrete various ECM molecules such as laminin, fibronectin, and the aforementioned Tenascin-C and CSPGs. It has been shown that the expression of laminin, fibronectin and vimentin in the injured SC can be significantly reduced by **siRNA targeting connective tissue growth factor (CTGF)**. This results in diminished glial scar formation, decreased inflammation and neuronal apoptosis, improving functional recovery in rats post-SCI [30].

Reducing excessive astrocyte proliferation and massive glial scar formation post-SCI might improve the chances for axonal regrowth and functional recovery. Polo-like kinase 4 (PLK4)’s expression increases significantly in astrocytes after SCI, peaking within a week of injury and supposedly promoting their proliferation. Local **RNAi of PLK4** in the injured SC reduced astrocyte proliferation and inflammatory responses while improving motor function recovery [31]. Furthermore, Smith et al. developed and applied in mice, with moderate contusion SCI, so-called three-way junction (3WJ) siRNAs targeting Lipocalin 2, GFAP and Vimentin—major effectors of astrocyte reactivity (reactive astroglyosis) [32]. Their work has demonstrated that such RNAi delivery platforms can be used both in vitro and in vivo to deliver siRNAs to reactive astrocytes and downregulate their targets. It has been suggested that Lipocalin 2 is a mediator of the rejection of neural transplants [33]. Thus, it is not surprising that reducing **Lipocalin 2 levels via RNAi** helped to establish a more suitable microenvironment for the transplantation of induced neural stem cells (iNSCs) in SCI [34]. Another molecule, old astrocyte specifically induced substance (OASIS), is a sensor and transducer of endoplasmic reticulum (ER) stress, upregulated by SCI. The **RNAi of OASIS** is linked to reduced astrogliosis and enhanced hindlimb motor function restoration [35]. SH3 domain-containing protein 1 (SASH1) maintains astrocytic differentiation. In cultured spinal astrocytes, the **RNAi of SASH1** led to decreased levels of secreted interferon-γ and elevated levels of secreted BDNF. Axonal growth and expression of the BDNF receptor (especially in axonal tips) were increased in cells co-cultured with astrocytes subjected to SASH1 RNAi. In a rat model of SCI, the RNAi of SASH1 facilitated functional recovery and hindered glial activation [36].

The formation of the astroglial-fibrotic scar after SCI is partially driven by the interaction between receptor EphB2 on fibroblasts and its ligand ephrin-B2 on astrocytes, which are both upregulated at the very beginning of SCI [37]. This interaction triggers the aggregation of these cells and deposition of ECM molecules like aggrecan and versican, which inhibit axonal growth [38]. In experiments utilizing the co-culture of astrocytes and fibroblasts and treatment with Transforming Growth Factor beta 1 (TGF-β1) to induce scar formation, **RNAi targeting ephrin-B2** decreased levels of aggrecan and versican and led to the formation of longer SC axons outgrowing through the scar (as assessed in vitro in a microfluidic platform) [38]. The fibrotic scar, formed by fibroblasts and components of the ECM adjacent to the astrocytic scar, is also a prominent feature of SCI in mammals, shaping lesion area and limiting neuroregeneration. Additional molecular targets involved in the secondary injury cascade and astroglial scar formation are a broad array of cytokines and growth factors. For example, the aforementioned TGF-β1 induces astrocyte and fibroblast activation contributing to scar formation after SCI [39]. While TGF-β1 can have neuroprotective effects, including on neural progenitor cells derived from SC [40], the overactivation of its downstream targets exacerbates astrogliosis, thus impeding regeneration. The RNAi of TGF -β1 might be one of the approaches to SCI therapy yet to be explored.

### 3.2. Inflammation and Immune Response

After the immediate injury, neuroinflammation becomes a central feature of SCI pathology. Immune cells, including microglia and blood-derived macrophages, infiltrate the injury site. Microglia cells shift towards a pro-inflammatory state, releasing cytokines that exacerbate inflammation and neuronal damage. Blood-borne macrophages follow, initially contributing to debris clearance; they eventually form foam cells that promote secondary neurodegeneration. Pro-inflammatory cytokines, such as Tumore Necrosis Factor-alpha (TNFα) and Interleukin-1 beta (IL-1β), are secreted, promoting further damage. However, anti-inflammatory responses also emerge, driven by alternatively activated anti-inflammatory and pro-regeneration (regulatory) M2 macrophages that attempt to repair tissue and limit the injury area. Numbers of M2 macrophages typically peak around three to seven days post-SCI, and then decline sharply [41]. On the contrary, pro-inflammatory M1 macrophages remain present in the lesion for a relatively long time, due to the influence of the microenvironment such as myelin debris. Several studies have demonstrated that the RNAi in SCI can reduce inflammatory responses, leading to decreased tissue damage and improved functional recovery. For example, in microglia and macrophages, the **silencing of interferon regulatory factor 5 (IRF5)**, a transcription factor known to upregulate sets of genes associated with the M1 macrophages phenotype, significantly reduced pro-inflammatory cytokines and improved functional recovery after SCI [42]. Similarly, **RNAi targeting IL-1β** promoted functional recovery in the rat contusion model of SCI [43]. Human antigen R (HuR) RNA-binding protein translocates to the cytoplasm in astrocytes after SCI and positively regulates the production of inflammatory cytokines such as TNF-α and IL-1β. The **knockdown of the HuR** in astrocytes decreased the expression of inflammatory cytokines, thereby potentially limiting secondary injury [44]. In a recent study by Gao et al., the **macrophage migration inhibitory factor (MIF)-targeted siRNA** co-administered with glial cell line-derived neurotrophic factor (GDNF) inhibited post-injury inflammation by facilitating macrophage M2 polarization [45].

The JAK/STAT3 pathway is a central signaling cascade responsible for initiating and sustaining astrocyte reactivity, activated by several cytokines and growth factors [46]; it also has neuron-specific functions, for example, its inhibition led to reduced apoptosis of neurons in a rat model of white matter injury [47]. The role of this pathway in SCI has been comprehensively reviewed recently [48]. Overall, the activation of this pathway might be pro- or anti-apoptotic depending on the context and cell type.

DNA released from dead cells (extracellular DNA, eDNA) also acts as a DAMP and triggers and amplifies neuroinflammation by activating immune cells. This eDNA forms structures known as neutrophil and macrophage extracellular traps (NETs and METs), which exacerbate secondary injury via inflammatory signaling pathways such as LL37 peptide/ P2X purinoceptor 7 receptor (P2X7R)/ nuclear factor kappa-light-chain-enhancer of the activated B cells (NF-κB) pathway [49]. Transcriptional factor NF-κB, inducing transcription of numerous pro-inflammatory genes including those encoding cytokines, such as TNF-α, IL-1β, Interleukin-6 (IL-6), etc., is a key modulator of inflammatory response in SCI. NF-κB inhibition confers neuroprotection by attenuating neuronal apoptosis and promoting the M2 microglial phenotype [50]. In the context of RNAi therapy, possible targets are NF-κB subunits (e.g., p65/RelA) or upstream kinases such as Inhibitor of κB kinase subunit beta (IKKβ) involved in NF-κB activation and nuclear translocation. The major sensor of eDNA is Stimulator of interferon genes (STING), also known as Transmembrane protein 173 (TMEM173), predominantly expressed by microglia. STING and NF-κB pathways are closely interconnected. In a mouse model of SCI, knockout of the gene encoding STING reduced the inflammatory response and promoted functional recovery by inhibiting NF-κB and MAPK signaling pathways [51]. Thus, the RNAi of STING might become a therapeutic strategy for SCI. Here, we stress that, at the same time, neuroinflammation has dual role in CNS injury and might promote axonal regeneration [52].

### 3.3. Cell Death and Senescence

Apoptosis is a key pathway leading to the death of neurons and oligodendrocytes after SCI, although other types of cell death are also evoked, such as necrosis, autophagy [53], necroptosis [54], ferroptosis [55], cuproptosis [56], pyroptosis [57], parthanatos [58], and others (summarized in [59]). SCI-associated apoptosis can be executed via the intrinsic (mitochondrial) and extrinsic (death receptor regulated) pathways [59]. Notably, in so-called “regenerating species” (e.g., zebrafish and axolotl, capable of regeneration of completely damaged SC), SCI-associated apoptosis is predominantly transient, peaking between around 24 h post-injury and declining sharply after ~5 days [60]. In non-regenerating species (e.g., rodents, humans), it peaks 24–48 h after injury and can persist for weeks [61,62]. Here, we leave behind other types of cell death in SCI and emphasize that there is an intricate interplay between different types of cell death after CNS trauma, and the inhibition (or stimulation) of one of them might affect others [63]. Furthermore, cell death is an integral part of the wound healing and regeneration process. Thus, perhaps the therapeutic approach to SCI cannot be as simplistic as the inhibition of cell death pathways.

Apart from cell death, cell senescence has emerged as an important contributor to secondary injury in SCI. Senescence is an irreversible cell cycle arrest accompanied by changes in gene expression, cell morphology, and secretion of pro-inflammatory factors (senescence-associated secretory phenotype, SASP). The aforementioned **IL-1β and TNF-α**, which can be targeted by RNAi, are SASP molecules, for example, it has been demonstrated that the **RNAi of IL-1β** leads to improved restoration of locomotor functions and reduced neuronal damage in the rat model of SCI [4]. Notably, TGF-β1 is released from activated macrophages after SCI triggers cellular senescence in neurons via SMAD2 signaling, thus contributing to impaired neural regeneration and functional recovery [64].

As discussed above, the clearance of senescent cells via apoptosis is beneficial, as it decreases pro-inflammatory SASP signaling. Thus, the inhibition of apoptosis as a therapeutic approach might be a “dual-edged sword”; perhaps it should be executed alongside approaches to decrease paracrine and autocrine inflammatory signaling of senescent cells, as described above.

RNAi might also have potential relevance in addressing glutamate excitotoxicity in the context of SCI—a pathological process where excessive glutamate overstimulates ionotropic glutamate receptors (iGluRs), leading to neuronal damage and death. Still, RNAi to silence excitotoxicity-related genes in SCI has not been explored yet. The **RNAi of inducible nitric oxide synthase (iNOS)** using siRNA-loaded chitosan nanoparticles, specifically directed at M1 macrophages, effectively decreased nitric oxide-mediated cell death associated with SCI [42].

### 3.4. Axonal Growth, Guidance and Axonal Transport

Molecular pathways determining axon growth after injury include several key ones, namely Phosphatase and tensin homolog/mechanistic target of rapamycin (**PTEN/mTOR**) signaling, **RhoA/Rho kinase (ROCK)** pathway signaling (discussed in detail in next sub-chapter), and others [65,66].

Based on the key roles of these pathways in axonal growth and guidance, as well as in several other aspects of SCI pathophysiology, a promising possible target of RNAi in SCI is **PTEN**, which negatively regulates the **PI3K/Akt/mTOR pathway.** This pathway regulates axon and dendrite growth, dendritic branching, synapse formation, and both the establishment and maintenance of neuronal polarity [67]. Silencing PTEN can enhance regenerative signaling, which is crucial for axonal growth. Experimental results show that the **siRNA-mediated knockdown of PTEN** promotes some improvement in animal models of SCI. For example, it improved axon regeneration [68] and tissue repair after SCI [69].

Notably, AKT regulates protein synthesis by acting on the downstream **GSK-3β/CCND2 pathway**, where AKT phosphorylates and inhibits GSK-3β, leading to the promotion of protein synthesis [70]. GSK-3β as a possible target of RNAi is discussed further in the text. Proper timing is of crucial importance in the case of targeting PI3K/Akt signaling. In the sub-acute phase after SCI, activation of the PI3K/Akt pathway helps inhibit inflammation and apoptosis, promoting neuronal survival and functional recovery. Thus, the negative regulation or inhibition of this pathway at this stage can worsen injury outcomes by allowing excessive cell death and inflammation [71]. On the other hand, in the chronic phase, sustained activation of PI3K/Akt/mTOR signaling promotes glial scar formation [71].

### 3.5. Cytoskeletal Dynamics

Cytoskeletal reorganization is essential for successful axon regeneration following CNS injury. The feasibility of targeting cytoskeletal proteins (microtubules, actin microfilaments, and neurofilaments) via RNAi as a therapy for SCI is indirectly supported by several studies utilizing small molecule inhibitors and activators of these proteins.

RhoA is a small GTPase that mediates growth cone collapse and inhibits axon regrowth, through the **RhoA/ROCK signaling pathway** promoting actin cytoskeleton contraction. The targeting of this pathway is under investigation in the context of SCI therapy. For example, VX-210 (BA-210/Cethrin), an inhibitor of ROCK, was investigated in clinical trial for SCI (SPRING trial, NCT02669849; it did not meet its primary efficacy endpoint). Overall, it has been shown that activation of the RhoA/ROCK pathway in SCI plays role in neuroinflammation, neuronal apoptosis, blood–brain barrier dysfunction, astrogliosis, and axon growth inhibition [66,72] whereas **silencing RhoA via siRNA** can promote neuronal survival and axon regeneration [73]. Interestingly, **miR-133b** (an endogenous miRNA) reduces RhoA expression and promotes neurite outgrowth. It has been shown that this delivery in combination with Argonaute-2 enhances recovery of the injured SC in mice with cervical contusion [74]. In another study, **RNAi of RhoA** was associated with a decrease in astrogliosis, apoptosis, smaller cavity size, and facilitated axonal regeneration [75]. The RhoA-mediated regulation of axonal projection and neuronal migration is also regulated by Slit2—an ECM glycoprotein secreted by glial cells. It exerts its effects by binding to transmembrane Roundabout (Robo) receptors expressed on various cell types in CNS. When Slit2 binds to Robo, it triggers intracellular signaling that increases RhoA activity. **RNAi of Robo1 receptor** resulted in the improvement in locomotor performance after SCI [76].

A novel therapeutic strategy for SCI is the **RNAi of Fidgetin-like 2 protein (FL2)**. FL2 is a microtubule-severing enzyme, negatively regulating axonal growth [77]. Following SCI, FL2 severs microtubules at the axonal growth cone, impeding axonal extension and reformation of the neuronal network. This disrupts axonal guidance and cytoskeletal reorganization. It has been demonstrated that the **RNAi of FL2** enhances microtubule stability, promotes axonal regeneration, and improves functional recovery in a rat’s cavernous nerve injury model [78]. Finally, the recent study by Smith et al. demonstrated that FL2 levels are markedly elevated after SCI in rats; the **RNAi of FL2** lead to improved locomotor function and partial preservation of corticospinal tract integrity [79]. Interestingly, it also led to an accumulation of microglia at the lesion site and increase in inflammatory cytokines IL-1β, TGF-β1, raising a question of whether combinatory treatment with corresponding RNAi might be beneficial.

Cyclin-dependent kinase 5 (CDK5) is predominantly expressed in neurons and regulates cytoskeletal dynamics crucial for axon growth and regeneration, via phosphorylating key cytoskeletal proteins such as neurofilament heavy chain, tau protein, and others [80]. Notably, it can both promote and inhibit growth cone stability affecting microtubule assembly and growth cone collapse. It is aberrantly activated in SC after injury and plays a significant role in the development of neurodegeneration associated with SCI. In particular, the complex of CDK5 and its activator p25 become hyperactive and mislocalized, leading to the phosphorylation of non-physiological substrates that promote neurotoxicity. Although direct studies on the RNAi-mediated knockdown of the gene encoding CDK5 specifically in SCI models are limited, research in related neurological injury models (reviewed in [81]) provides a rationale for assessment of its value as a therapeutic target. As outlined in the aforementioned review by Ao C. et al., the **RNAi of CDK5** in ischemic brain injury models prevents neurodegeneration, reduces glial activation, and improves motor and cognitive outcomes, suggesting neuroprotective and anti-inflammatory effects that might also be beneficial in SCI—subject to further tests. Finally, the aforementioned GSK3β regulates cytoskeletal assembly in axon growth cones [82] and its RNAi promotes neurite/axon growth in retinal ganglion [83]. No data exists yet on the role of GSK3β RNAi in SCI.

### 3.6. Mitochondrial Dysfunction

In SCI, mitochondrial dysfunction is a key barrier to regeneration due to the energy-intensive nature of axonal growth. A recent investigation by Han et al. presented a novel approach to SCI treatment through the **RNAi of syntaphilin (SNPH)**, a mitochondrial anchor protein, to restore cellular energetics. SNPH binds mitochondria to the cytoskeleton, preventing their movement to areas of the axon where energy demand is highest, such as the growth cone. This contributes to mitochondrial dysfunction post-injury and hinders axonal regeneration. The depletion of SNPH in mice enhanced mitochondrial transport, supposedly promoting the clearance of damaged mitochondria, as well as improving the energy supply to injured axons and promoting axonal regeneration. This resulted in partial functional recovery in the mouse model of SCI [84]. Axons in *Snph*-deficient mice exhibited increased mitochondrial integrity, reduced reactive oxygen species (ROS) production, and extended axonal growth beyond the injury site. Further, **RNAi of SNPH** led to increased mitochondrial mobility, reduced mitochondrial stress, and enhanced axonal regeneration in the rodent SCI model. Mice treated with shRNA targeting SNPH displayed better functional recovery and increased axonal growth across the injury site compared to controls. Additionally, this study highlighted that enhancing mitochondrial bioenergetics could not only support axonal regeneration but also protect neurons from further degeneration [85]. In vivo, **RNAi of SNPH** led to improved neurobehavioral outcomes in mice subjected to intracerebral hemorrhage. The findings suggest that targeting SNPH might become a therapeutic strategy for SCI as well [86].

### 3.7. Increased Blood–Spinal Cord Barrier (BSCB) Permeability

Pathologically increased blood–spinal cord barrier (BSCB) permeability following SCI results from direct vascular damage and the disruption of tight junctions between endothelial cells. This abnormal permeability occurs rapidly after injury, with an initial peak within hours and secondary peak days later (during revascularization), resulting in the infiltration of immune cells and inflammatory mediators that exacerbate secondary injury processes [87]. The disruption of BSCB can persist for weeks, impairing tissue repair and functional recovery. Restoring BSCB integrity is therefore critical for limiting secondary damage and improving outcomes after SCI. Transient Receptor Potential Melastatin 7 (TRPM7), a bi-functional protein acting as a cation channel and a kinase, is upregulated after SCI and contributes to BSCB disruption [88]. Pharmacological targeting of TRPM7 with carvacrol mitigates BSCB damage and leads to functional improvement [89]. The clinical utility of RNAi targeting TRPM7 in SCI is yet to be tested.

## 4. Combination of RNAi and Other SCI Therapies

Numerous clinical studies show that the transplantation of Mesenchymal stem cells (MSCs) or treatment with MSC-derived extracellular vesicles (EVs) can improve sensory and motor function post-SCI. This is attributed to their anti-inflammatory and neuroprotective properties, mainly resulting from their ability to secrete an array of biologically active molecules. The molecular cargo of EVs secreted by MSCs is known to contain miRNAs targeting biomolecules which are involved in many pathological processes discussed above. For example, PTEN is targeted by miR-29b-3p in MSCs-secreted EVs [90]. There is a body of literature illustrating attempts to use cell therapy by MSCs in combination with the RNAi. Given their very low immunogenicity, MSCs are not rejected after transplantation and, apart from their roles discussed above, can also serve as vehicles for the delivery of therapeutic interfering RNAs. For example, MSCs-derived exosomes were used for the delivery of siRNA targeting CTGF in a rat model of SCI resulting in locomotor improvement [30]. Here, we provide a couple more examples of such combinational approaches. The **RNAi of Nogo-66 receptor (NgR)** in bone marrow-derived MSCs before transplantation promoted nerve regeneration, enhanced behavioral recovery, and decreased mortality rates in rats with SCI, compared to control group (transplantation of MSCs only) [91]. Cyclin D1, a key cell cycle regulator, is abnormally upregulated after SCI, causing neurons and glial cells to re-enter the cell cycle. Notably, so-called abortive cell cycle re-entry leads to programmed cell death of most-mitotic neurons. Speaking of glial cells, Cyclin D1 upregulation is linked to their activation and hence neuroinflammation, which contributes to secondary injury and chronic neuropathic pain after SCI [92]. In a rat model, SCI transplantation of bone marrow-derived MSCs (BMSCs) with plasmid expressing **Cyclin D1 siRNA** led to higher degree of SC tissue repair compared to animals receiving BMSCs without siRNA [93]. It remains to be investigated whether the impact of siRNA in this case was mostly due to the downregulation of Cyclin D1 in BMSCs or because of the secretion of BMSCs-derived EVs with siRNA, resulting in the downregulation of Cyclin D1 in adjacent glial cells and neurons. As mentioned, not only MSCs but also MSC-derived EVs might be used as SCI therapy. The potential of such an approach was demonstrated in a rat model of complete SCI, where siRNA targeting PTEN was delivered intranasally in MSCs-derived exosomes, facilitating tissue repair [69]. Apart from MSC, other cells, such as neural stem/progenitor cells (NSPCs) and directly reprogrammed neural progenitor cells, hold great translational potential for SCI treatment, as was demonstrated in numerous studies including our recent work in primates [94]. Leaving behind the topic of the NSPCs’ transplantation, it can be said that the **RNAi of protein 21 (p21)** can boost proliferation of endogenous NSPCs in SCI. In particular, it has been demonstrated recently that application of a cationic liposome-based delivery system carrying p21 siRNA in vitro leads to the increased proliferation rate of primary NSPC, with no effect on their differentiation capacity. In vivo, in the rat model of T9 SCI, a combination treatment with gelatin hydrogels incorporating this RNAi stimulated NSPCs proliferation and migration at SCI lesion sites, as well as aided dense tissue cable formation. Ultimately, this resulted in accelerated locomotor function recovery [95].

Biomaterial-based curative strategies, including the use of aforementioned hydrogels and other biomaterials, alone or in combination with other treatments, are on a rise in the field of SCI treatment [96]. Here, we provide a couple of examples of combining RNAi and biomaterials. Promising results were obtained using locally administered photocurable lipid nanoparticle GelMA (PLNG) hydrogel scaffold, allowing for controlled delivery at the SCI lesion area of **dual siRNA simultaneously targeting PTEN and MIF.** The application of this scaffold created a local anti-inflammatory environment promoting neural repair (which led to an increase in growth factors and decrease in pro-inflammatory factors) [97]. Based on the fact that the protein encoded by immune checkpoint gene Tim3 contributes to excessive inflammatory response in SCI and is highly expressed in microglia, the system was created combining **Tim3 siRNA** delivered by exosomes encapsulated within a hydrogel (RNAi-Tim3-Exo@SF hydrogel system). In a mouse model of SCI, this system had the potential to promote damaged axonal regeneration, modulate inflammation at the lesion site, promote angiogenesis and stabilize microtubules, and ultimately improve motor functions [98].

## 5. Discussion

**Figure 2:**
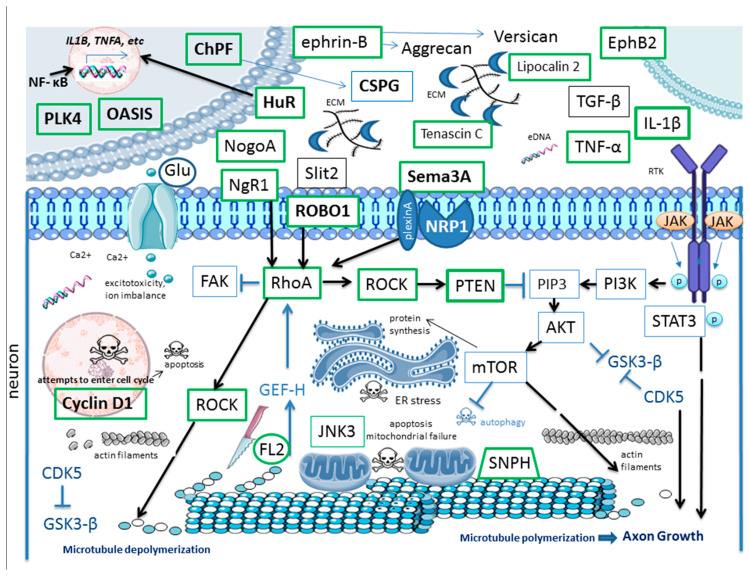
Selected pathways and RNAi targets in the context of SCI therapy. Known RNAi targets are shown in green boxes. Activation is depicted by arrows; inhibition is depicted by T-bar. All abbreviations are defined in the list of abbreviations; description of pathways and corresponding references are provided in the manuscript. The figure was designed and created using Biorender (accessed on 22 June 2025), Server Medical Art (accessed on 2 March 2024, licensed under a Creative Common Attribution 3.0 Unported License; https://smart.servier.com (accessed on 2 March 2024)) and Bioicons (accessed on 2 March 2024, licensed under a Creative Common license; https://bioicons.com (accessed on 2 March 2024)).

Here, we provided a broad overview of the current targets of RNAi in the context of SCI therapy and corresponding key pathways (Figure 2, Supplementary Table S1). This list is not complete and is expected to grow.

It should be noted that there are several common types of SCI and, reflecting this, several types of experimental SCI models, namely contusion, transection, compression, distraction, dislocation, or chemical models of SCI exist. Contusion models mimic blunt trauma, transection models are based on partial or complete rupture of the SC, compression models replicate sustained occlusion of the SC, distraction models utilize controlled stretching of the SC, dislocation models recapitulate lateral displacement of vertebra resulting in SCI, and chemical models focus on secondary damage caused by trauma (excitotoxicity, etc.) [99,100]. Furthermore, different sites of injury, the force of impact, and different animal models can be used. Thus, anatomical, physiological, histological, molecular and cellular features of these models vary, and results obtained in one model may not be necessarily reproduced in others. Furthermore, some targets identified in animal models of SCI may not have translational value, given the well-known difference between the molecular mechanisms of SC regeneration in humans and in rodents (animals commonly used to model SCI).

There are several hurdles that must be overcome to bring RNAi “from bench to bedside”. Perhaps a central obstacle to RNAi-based SCI therapy is the stability–potency tradeoff: unmodified si/shRNAs are rapidly degraded and cleared, whereas protective modifications might hinder the loading of RNA-induced silencing complex (RISC), perturb si/shRNA trafficking, or change their biodistribution. Aforementioned, there are several types of RNAi and various delivery systems of RNAi; each of them has its own advantages and disadvantages, and many approaches can be used in attempt to overcome the challenges associated with their use, comprehensively reviewed elsewhere [101,102,103]. As for the challenges, to name a few, microglia and macrophages which infiltrate the lesion area avidly scavenge lipid nanoparticles (LNP) with siRNA cargo, diverting them from target cells—neurons and oligodendrocytes—and biasing knockdown toward glial/immune cells. The targeted delivery of RNAi in the case of SCI is further constrained by the dense ECM and glial scar that impede diffusion and electrostatically sequester polycationic carriers of RNAi. Even after sufficient cellular uptake of RNAi within LNP, most of it is routed to lysosomes, so various strategies should be applied to facilitate cytosolic rather than lysosomal delivery of RNAi. Too much of RNAi drugs might trigger off-target RNAi-driven silencing, leading to transcriptome-wide dysregulation of gene expression. This is particularly detrimental in spinal circuits where small shifts in gene expression (and, subsequently, excitability) can provoke pain or spasticity. Perhaps, when it comes to RNAi as a molecular tool, too much inhibition (i.e., too many targets, or for too long period of time, or applied too early, etc.) is as bad as no inhibition, and the best strategy is “everything in moderation”, aimed at keeping a balance between beneficial and detrimental responses to SCI. For example, reactive astrogliosis is a part of “a healing response”, whereas, if excessive, its adverse effects make a glial scar. Notably, given the possible off-target effects, perhaps results obtained in experiments using RNAi should be “taken with a grain of salt” and validated using different approaches [104]. The patient-specific therapeutic window for RNAi is challenging to determine, especially given the dual, often opposite role of some targets depending on the stage of SCI and cellular context. Considering the intricate time-dependent dynamics of cellular and molecular responses in SCI, precise control over the timing and dosage of RNAi treatment is critical to achieving therapeutic effectiveness. Finally, RNAi can trigger innate immunity, amplifying secondary injury [105].

The “fine-tuned”, targeted RNAi-based approach, taking into account all nuances of response to SCI of particular cell-subpopulations, might be more appropriate. For example, hypothetically, rather than inhibiting apoptosis in all cells, the better approach would be to target particular cell-specific apoptosis-regulating pathways and rescue some cells while letting other types of cells at the lesion site die (to allow for axonal growth, etc.). Given the dual and cell type-specific role in SCI of some aforementioned targets, it is advisable rather than delivering RNAi molecules to all cells at the lesion site, to use a cell-specific approach. Genetic constructs, including those encoding therapeutic shRNA molecules, can be designed to be exclusively transcribed in particular types of cells. This might be achieved by use of cell type-specific promoters, for example, astrocyte-specific GfaABC1D promoter, neuron-specific promoters of synapsin and CamKIIα, microglia-specific promoter Iba1, or oligodendrocyte-specific promoters of genes encoding myelin basic protein and proteolipid protein in combination with the “miRNA-embedding” or “miRNA scaffolding” approach (incorporation of shRNA sequence into flanking regions of natural miRNAs). Such constructs can be used for cell-specific RNAi, including the RNAi of multiple targets. Additionally, RNAi drugs can be modified for selective uptake to particular types of cells, by conjugating interfering RNAs or nanoparticles containing these RNAs to ligands or antibodies that recognize cell-specific surface receptors, enabling receptor-mediated endocytosis and gene silencing exclusively in target cells. An example of such an approach is astrocyte-targeted delivery of siRNA using adenosine-functionalized lipid nanoparticles (Ad4 LNPs) in a mouse model of traumatic brain injury (TBI) [106].

RNAi might have a translational potential not as a single therapy for SCI, but rather as a complementary one to the current and emerging therapies for SCI (such as cell therapy, use of bio-active molecules, tissue scaffolds, etc., alone or in combination) which to some degree might be enhanced by RNAi. For example, cell therapy with transplanted MSCs exerts anti-inflammatory effects in SCI in part by inhibiting the Jagged1/Notch signaling pathway. RNAi targeting Jagged1 also decreases SCI-induced inflammation and increases the survival of neurons [107]. Perhaps using both approaches might be more efficient.

Of particular interest are novel RNAi tools, such as CRISPR-Cas13 RNA-targeting systems capable of precise RNAi in mammalian cells, given that their mechanism of action differs from other aforementioned molecular tools of RNAi. It has been shown recently that such systems can be used in the nervous system (including SC), leading to up to 50 percent reduction in some target proteins [108].

Notably, not only protein-coding genes but regulatory long non-coding RNAs (lncRNAs) are suitable targets for RNAi in SCI, as it has been demonstrated in several studies. For example, in a rat model of acute SCI and hypoxic cell model, the **knockdown of BDNF-antisense lncRNA (BDNS-AS)** using siRNA suppressed apoptosis in neurons [109]. In a complete transection at the T10 rat model of SCI, the **RNAi of LncRNA Vof-16** promoted nerve regeneration and functional recovery [110]. Other lncRNA targets for RNAi in SCI have also been identified [111,112].

## 6. Conclusions

In this study, we overviewed the principal pathogenetic mechanisms of SCI and molecular targets for RNAi therapy involved in those mechanisms, such as molecules participating in ECM remodeling and scar formation, inflammatory and immune responses, programmed cell death and cellular senescence, axonal growth and guidance, axonal transport, cytoskeletal dynamics, mitochondrial dysfunction, and others. These insights provide a foundation for a systemic and informed approach to RNAi-based therapeutics in SCI. It is important to acknowledge that further investigations are needed, in different models of SCI, aimed at determining a sequence of molecular and cellular responses to trauma at the single-cell resolution, which will aid the development of cell-specific tailored RNAi panels with enhanced therapeutic efficacy. We also point out that the combination of RNAi with other therapies (for example, biomaterial-based) have a significant translational potential, as evidenced by several publications. Furthermore, not only protein-coding genes but also non-coding RNAs are suitable targets for RNAi. Finally, novel RNAi tools such as CRISPR-Cas13 might revolutionize the field and offer new opportunities for the development of RNAi-based therapies.

## Data Availability

Not applicable.

## Supplementary Materials

The following supporting information can be downloaded at: https://www.mdpi.com/article/10.3390/ijms26209861/s1.

## Abbreviations

3WJ	Three-Way Junction (nanoparticles)
AAV	Adeno-Associated Virus
Ad4 LNPs	Adenosine-functionalized Lipid Nanoparticles
ADSCs	Adipose-Derived Stem Cells
Ago2	Argonaute-2 Protein
anti-RGMa Ab	Antibody to RGMa
ASO	Single-stranded antisense RNA oligonucleotides
BBB	Blood–Brain Barrier
BDNF	Brain-Derived Neurotrophic Factor
BMSCs	Bone Marrow-Derived Mesenchymal Stem Cells
BSCB	Blood–Spinal Cord Barrier
CCL2	Chemokine (C-C Motif) Ligand 2
CD206	Cluster of Differentiation 206 (Macrophage Mannose receptor)
CD86	Cluster of Differentiation 86
CDK5	Cyclin dependent kinase 5
ChABC	Chondroitinase ABC
ChPF	Chondroitin Polymerizing Factor
CNS	Central Nervous System
Col1a1	Collagen Type I Alpha 1 Chain
CREB3L1	cAMP-Responsive Element-Binding Protein 3-Like 1 (OASIS)
CSPGs	Chondroitin Sulfate Proteoglycans
CTGF	Connective Tissue Growth Factor
CX3CR1	Chemokine Receptor 1
DAMPs	Damage-Associated Molecular Patterns
ECM	Extracellular Matrix
EphB2	Ephrin Receptor B2
ESCs	Embryonic stem cells
EVs	Extracellular Vesicles
FDX1	Ferredoxin 1
FL2	Fidgetin-Like 2 Protein
GAP-43	Growth Associated Protein 43
GelMA	Gelatin Methacrylate
GFAP	Glial Fibrillary Acidic Protein
GDNF	Glial Cell-Derived Neurotrophic Factor
GiPCRs	G Protein-Coupled Receptors
GSK-3β	Glycogen Synthase Kinase-3β
hATTR	Hereditary Transthyretin-mediated amyloidosis
HucMSCs	Human umbilical cord Mesenchymal Stem Cells
HuR	Hu Antigen R
IBA1	Ionized Calcium Binding Adaptor Molecule 1
ICH	Intracerebral Hemorrhage
IKKβ	Inhibitor of κB kinase subunit beta
IL-1β	Interleukin 1 Beta
IL-6	Interleukin 6
IL-10	Interleukin 10
IL-12	Interleukin 12
iGluRs	Ionotropic Glutamate Receptors
iNSCs	Induced Neural Stem Cells
iNOS	Inducible Nitric Oxide Synthase
iPSCs	Induced pluripotent stem cells
IRF5	Interferon Regulatory Factor 5
JAK	Janus Kinase
Lcn2	Lipocalin 2
LNPs	Lipid Nanoparticles
LV	Lentiviral Vector
MAG	Myelin-Associated Glycoprotein
MAI	Myelin-Associated Inhibitor
MCAO	Middle Cerebral Artery Occlusion
METs	Macrophage Extracellular traps
MIF	Macrophage Migration Inhibitory Factor
miRNA	MicroRNA
mRNA	Messenger RNA
mTOR	Mechanistic Target of Rapamycin
MSCs	Mesenchymal Stem Cells
NETs	Neutrophil Extracellular traps
NF-κB	Nuclear Factor Kappa-light-chain-enhancer of activated B cells
NeuN	Neuronal Nuclei Marker
NgR	Nogo-66 Receptor
Nogo-A	Neurite Outgrowth Inhibitor A
NSCs	Neural Stem Cells
OASIS	Old Astrocyte Specifically Induced Substance
OMgp	Oligodendrocyte Myelin Glycoprotein
P2X7R	P2X purinoceptor 7 receptor
PARP-1	Poly(ADP-Ribose) Polymerase 1
PBS	Phosphate-buffered Saline
PI3K	Phosphoinositide 3-Kinase
PLK4	Polo-Like Kinase 4
PLNG	Photocurable Lipid Nanoparticle GelMA
PMCID / PMID	PubMed Central Identification Number/ PubMed Identifier
PNNs	Perineuronal Nets
PTEN	Phosphatase and Tensin Homolog
RAGE	Receptor for Advanced Glycation End Products
RGMA	Repulsive Guidance Molecule A
RhoA	Ras Homolog Family Member A
RNAi	RNA Interference
Robo	transmembrane Roundabout receptors
ROCK	Rho-Associated Protein Kinase
ROS	Reactive Oxygen Species
SASP	Senescence-Associated Secretory Phenotype
SC	Spinal Cord
SCI	Spinal Cord Injury
sem3A	Semaphorin 3A
shRNA	Short Hairpin RNA
siRNA	Small Interfering RNA
SNPH	Syntaphilin
STAT3	Signal Transducer and Activator of Transcription 3
STING	Stimulator of Interferon Genes
TBI	Traumatic Brain Injury
TGF-β1	Transforming Growth Factor Beta 1
TLR4	Toll-Like Receptor 4
TLRs	Toll-Like Receptors
TMEM119	Transmembrane Protein 119
TMEM173	Transmembrane Protein 173
TNF-α	Tumor Necrosis Factor Alpha
TNC	Tenascin-C
TRPM7	Transient Receptor Potential Melastatin 7
TRITC	Tetramethylrhodamine
VIM	Vimentin

## References

[1] Kang Y., Ding H., Zhou H., Wei Z., Liu L., Pan D., Feng S. (2017). Epidemiology of Worldwide Spinal Cord Injury: A Literature Review. J. Neurorestoratology.

[2] Hu X., Xu W., Ren Y., Wang Z., He X., Huang R., Ma B., Zhao J., Zhu R., Cheng L. (2023). Spinal Cord Injury: Molecular Mechanisms and Therapeutic Interventions. Signal Transduct. Target. Ther..

[3] Setten R.L., Rossi J.J., Han S.P. (2019). The Current State and Future Directions of RNAi-Based Therapeutics. Nat. Rev. Drug Discov..

[4] Lin J., Jo S.B., Kim T.H., Kim H.W., Chew S.Y. (2020). RNA Interference in Glial Cells for Nerve Injury Treatment. J. Tissue Eng..

[5] Yang W., Sun P. (2020). Promoting Functions of MicroRNA-29a/199B in Neurological Recovery in Rats with Spinal Cord Injury through Inhibition of the RGMA/STAT3 Axis. J. Orthop. Surg. Res..

[6] Zhou H.J., Wang L.Q., Xu Q.S., Fan Z.X., Zhu Y., Jiang H., Zheng X.J., Ma Y.H., Zhan R.Y. (2016). Downregulation of MiR-199b Promotes the Acute Spinal Cord Injury through IKKβ-NF-ΚB Signaling Pathway Activating Microglial Cells. Exp. Cell Res..

[7] Michael F.M., Chandran P., Chandramohan K., Iyer K., Jayaraj K., Sundaramoorthy R., Venkatachalam S. (2019). Prospects of SiRNA Cocktails as Tools for Modifying Multiple Gene Targets in the Injured Spinal Cord. Exp. Biol. Med..

[8] Lennox K.A., Behlke M.A. (2016). Cellular Localization of Long Non-Coding RNAs Affects Silencing by RNAi More than by Antisense Oligonucleotides. Nucleic Acids Res..

[9] O’Shea T.M., Burda J.E., Sofroniew M.V. (2017). Cell Biology of Spinal Cord Injury and Repair. J. Clin. Investig..

[10] Anjum A., Yazid M.D., Fauzi Daud M., Idris J., Ng A.M.H., Selvi Naicker A., Ismail O.H.R., Athi Kumar R.K., Lokanathan Y. (2020). Spinal Cord Injury: Pathophysiology, Multimolecular Interactions, and Underlying Recovery Mechanisms. Int. J. Mol. Sci..

[11] Grin A.A., Kordonskiy A.Y., Abdukhalikov B.A., Arakelyan S.L., Lvov I.S., Kaikov A.K., Talypov A.E., Sytnik A.V. (2021). Classification of Injuries of the Thoracic and Lumbar Spine. Russ. J. Neurosurg..

[12] Sánchez-Ventura J., Lane M.A., Udina E. (2022). The Role and Modulation of Spinal Perineuronal Nets in the Healthy and Injured Spinal Cord. Front. Cell Neurosci..

[13] Alastra G., Quadalti C., Baldassarro V.A., Giuliani A., Giardino L., Calzà L. (2025). The Influence of Pathological Extracellular Matrix on the Biological Properties of Stem Cells: Possible Hints for Cell Transplantation Therapies in Spinal Cord Injury. Int. J. Mol. Sci..

[14] Burnside E.R., Bradbury E.J. (2014). Review: Manipulating the Extracellular Matrix and Its Role in Brain and Spinal Cord Plasticity and Repair. Neuropathol. Appl. Neurobiol..

[15] Rolls A., Shechter R., London A., Segev Y., Jacob-Hirsch J., Amariglio N., Rechavi G., Schwartz M. (2008). Two Faces of Chondroitin Sulfate Proteoglycan in Spinal Cord Repair: A Role in Microglia/Macrophage Activation. PLoS Med..

[16] Yang D., Han Z., Oppenheim J.J. (2017). Alarmins and Immunity. Immunol. Rev..

[17] Francos-Quijorna I., Sánchez-Petidier M., Burnside E.R., Badea S.R., Torres-Espin A., Marshall L., de Winter F., Verhaagen J., Moreno-Manzano V., Bradbury E.J. (2022). Chondroitin Sulfate Proteoglycans Prevent Immune Cell Phenotypic Conversion and Inflammation Resolution via TLR4 in Rodent Models of Spinal Cord Injury. Nat. Commun..

[18] Chambel S.S., Cruz C.D. (2023). Axonal Growth Inhibitors and Their Receptors in Spinal Cord Injury: From Biology to Clinical Translation. Neural Regen. Res..

[19] Laabs T.L., Wang H., Katagiri Y., McCann T., Fawcett J.W., Geller H.M. (2007). Inhibiting Glycosaminoglycan Chain Polymerization Decreases the Inhibitory Activity of Astrocyte-Derived Chondroitin Sulfate Proteoglycans. J. Neurosci..

[20] Didangelos A., Puglia M., Iberl M., Sanchez-Bellot C., Roschitzki B., Bradbury E.J. (2016). High-Throughput Proteomics Reveal Alarmins as Amplifiers of Tissue Pathology and Inflammation after Spinal Cord Injury. Sci. Rep..

[21] Chelluboina B., Chokkalla A.K., Mehta S.L., Morris-Blanco K.C., Bathula S., Sankar S., Park J.S., Vemuganti R. (2022). Tenascin-C Induction Exacerbates Post-Stroke Brain Damage. J. Cereb. Blood Flow Metab..

[22] Stepankova K., Smejkalova B., Machova Urdzikova L., Haveliková K., de Winter F., Suchankova S., Verhaagen J., Herynek V., Turecek R., Kwok J. (2025). Activated Alpha 9 Integrin Expression Enables Sensory Pathway Reconstruction after Spinal Cord Injury. Acta Neuropathol. Commun..

[23] Hirt J., Khanteymoori A., Hohenhaus M., Kopp M.A., Howells D.W., Schwab J.M., Watzlawick R. (2023). Inhibition of the Nogo-Pathway in Experimental Spinal Cord Injury: A Meta-Analysis of 76 Experimental Treatments. Sci. Rep..

[24] Kucher K., Johns D., Maier D., Abel R., Badke A., Baron H., Thietje R., Casha S., Meindl R., Gomez-Mancilla B. (2018). First-in-Man Intrathecal Application of Neurite Growth-Promoting Anti-Nogo-A Antibodies in Acute Spinal Cord Injury. Neurorehabilit. Neural Repair.

[25] Wang J., Yang J., Ma Y., Hua Z., Guo Y., Gu X., Zhang Y. (2015). Nogo-A Expression Dynamically Varies after Spinal Cord Injury. Neural Regen. Res..

[26] Hata K., Fujitani M., Yasuda Y., Doya H., Saito T., Yamagishi S., Mueller B.K., Yamashita T. (2006). RGMa Inhibition Promotes Axonal Growth and Recovery after Spinal Cord Injury. J. Cell Biol..

[27] Qu Y., Zhao J., Wang Y., Gao Z. (2014). Silencing EphrinB3 Improves Functional Recovery Following Spinal Cord Injury. Mol. Med. Rep..

[28] Kim S.J., Ko W.K., Han G.H., Lee D., Cho M.J., Sheen S.H., Sohn S. (2023). Axon Guidance Gene-Targeted SiRNA Delivery System Improves Neural Stem Cell Transplantation Therapy after Spinal Cord Injury. Biomater. Res..

[29] Li H., Liu Y., Sun Y., Guo H., Lv S., Guo W., Ren J., Wang Y., Zu J., Yan J. (2024). Targeting Astrocytes Polarization after Spinal Cord Injury: A Promising Direction. Front. Cell Neurosci..

[30] Huang W., Qu M., Li L., Liu T., Lin M., Yu X. (2021). SiRNA in MSC-Derived Exosomes Silences CTGF Gene for Locomotor Recovery in Spinal Cord Injury Rats. Stem Cell Res. Ther..

[31] Gu Y., Zhang R., Jiang B., Xu X., Guan J.J., Jiang X.J., Zhou Y., Zhou Y.L., Chen X. (2022). Repair of Spinal Cord Injury by Inhibition of PLK4 Expression Through Local Delivery of SiRNA-Loaded Nanoparticles. J. Mol. Neurosci..

[32] Smith J.A., Braga A., Verheyen J., Basilico S., Bandiera S., Alfaro-Cervello C., Peruzzotti-Jametti L., Shu D., Haque F., Guo P. (2018). RNA Nanotherapeutics for the Amelioration of Astroglial Reactivity. Mol. Ther. Nucleic Acids.

[33] Weng Y.C., Huang Y.T., Chiang I.C., Tsai P.J., Su Y.W., Chou W.H. (2021). Lipocalin-2 Mediates the Rejection of Neural Transplants. FASEB J..

[34] Braga A., Bandiera S., Verheyen J., Hamel R., Rutigliani C., Edenhofer F., Smith J.A., Pluchino S. (2020). Combination of In Situ Lcn2 PRNA-RNAi Nanotherapeutics and INSC Transplantation Ameliorates Experimental SCI in Mice. Mol. Ther..

[35] Takazawa A., Kamei N., Adachi N., Ochi M. (2018). Endoplasmic Reticulum Stress Transducer Old Astrocyte Specifically Induced Substance Contributes to Astrogliosis after Spinal Cord Injury. Neural Regen. Res..

[36] Liu S., Lin G., Yang Q., Wang P., Ma C., Qian X., He X., Dong Z., Liu Y., Liu M. (2023). Depletion of SASH1, an Astrocyte Differentiation-related Gene, Contributes to Functional Recovery in Spinal Cord Injury. CNS Neurosci. Ther..

[37] Bundesen L.Q., Scheel T.A., Bregman B.S., Kromer L.F. (2003). Ephrin-B2 and EphB2 Regulation of Astrocyte-Meningeal Fibroblast Interactions in Response to Spinal Cord Lesions in Adult Rats. J. Neurosci..

[38] Li Y., Chen Y., Tan L., Pan J.Y., Lin W.W., Wu J., Hu W., Chen X., Wang X.D. (2017). RNAi-Mediated Ephrin-B2 Silencing Attenuates Astroglial-Fibrotic Scar Formation and Improves Spinal Cord Axon Growth. CNS Neurosci. Ther..

[39] Ma C., Wang Z., Ran R., Liao H., Lyu J., Ren Y., Lei Z., Zhang H. (2024). TGF-β Signaling Pathway in Spinal Cord Injury: Mechanisms and Therapeutic Potential. J. Neurosci. Res..

[40] Park S.M., Jung J.S., Jang M.S., Kang K.S., Kang S.K. (2008). Transforming Growth Factor-β1 Regulates the Fate of Cultured Spinal Cord-derived Neural Progenitor Cells. Cell Prolif..

[41] Wang X., Cao K., Sun X., Chen Y., Duan Z., Sun L., Guo L., Bai P., Sun D., Fan J. (2015). Macrophages in Spinal Cord Injury: Phenotypic and Functional Change from Exposure to Myelin Debris. Glia.

[42] Li J., Liu Y., Xu H., Fu Q. (2016). Nanoparticle-Delivered IRF5 SiRNA Facilitates M1 to M2 Transition, Reduces Demyelination and Neurofilament Loss, and Promotes Functional Recovery After Spinal Cord Injury in Mice. Inflammation.

[43] Cao J.-F., Hu X., Xiong L., Wu M., Yang X., Wang C., Chen S., Xu H., Chen H., Ma X. (2022). Interference of Interleukin-1 β Mediated by Lentivirus Promotes Functional Recovery of Spinal Cord Contusion Injury in Rats via the PI3K/AKT1 Signaling Pathway. Mediators Inflamm..

[44] Kwan T., Floyd C.L., Kim S., King P.H. (2017). RNA Binding Protein Human Antigen R Is Translocated in Astrocytes Following Spinal Cord Injury and Promotes the Inflammatory Response. J. Neurotrauma.

[45] Gao Y., Wang K., Wu Y., Wu S., Ma P., Zhang J., Li J., Shen G., Men K. (2025). Controlled Release of MIF SiRNA and GDNF Protein from a Photocurable Scaffold Efficiently Repairs Spinal Cord Injury. MedComm.

[46] Krebs D.L., Hilton D.J. (2001). SOCS Proteins: Negative Regulators of Cytokine Signaling. Stem Cells.

[47] Chen X.-M., Yu Y.-H., Wang L., Zhao X.-Y., Li J.-R. (2019). Effect of the JAK2/STAT3 Signaling Pathway on Nerve Cell Apoptosis in Rats with White Matter Injury. Eur. Rev. Med. Pharmacol. Sci..

[48] Guo X., Jiang C., Chen Z., Wang X., Hong F., Hao D. (2023). Regulation of the JAK/STAT Signaling Pathway in Spinal Cord Injury: An Updated Review. Front. Immunol..

[49] Zhang C., Guo D., Qiao H., Li J., Li J., Yang Y., Chang S., Li F., Wang D., Li H. (2022). Macrophage Extracellular Traps Exacerbate Secondary Spinal Cord Injury by Modulating Macrophage/Microglia Polarization via LL37/P2X7R/NF- κ B Signaling Pathway. Oxid. Med. Cell Longev..

[50] Varsamos I., Patilas C., Galanis A., Zachariou D., Tsalimas G., Sakellariou E., Spyrou I., Rozis M., Kaspiris A., Karampinas P.K. (2025). The Impact of Nuclear Factor Kappa B on the Response of Microglia in Spinal Cord Injuries. Cureus.

[51] Wang Y.Y., Shen D., Zhao L.J., Zeng N., Hu T.H. (2019). Sting Is a Critical Regulator of Spinal Cord Injury by Regulating Microglial Inflammation via Interacting with TBK1 in Mice. Biochem. Biophys. Res. Commun..

[52] Bollaerts I., Van Houcke J., Andries L., De Groef L., Moons L. (2017). Neuroinflammation as Fuel for Axonal Regeneration in the Injured Vertebrate Central Nervous System. Mediators Inflamm..

[53] Ribas V.T., Lingor P. (2015). Autophagy in Degenerating Axons Following Spinal Cord Injury: Evidence for Autophagosome Biogenesis in Retraction Bulbs. Neural Regen. Res..

[54] Hu X., Xu Y., Zhang H., Li Y., Wang X., Xu C., Ni W., Zhou K. (2022). Role of Necroptosis in Traumatic Brain and Spinal Cord Injuries. J. Adv. Res..

[55] Bai X.Y., Liu X.L., Deng Z.Z., Wei D.M., Zhang D., Xi H.L., Wang Q.Y., He M.Z., Yang Y.L. (2023). Ferroptosis Is a New Therapeutic Target for Spinal Cord Injury. Front. Neurosci..

[56] Zhou Y., Li X., Wang Z., Ng L., He R., Liu C., Liu G., Fan X., Mu X., Zhou Y. (2025). Machine Learning-Driven Prediction Model for Cuproptosis-Related Genes in Spinal Cord Injury: Construction and Experimental Validation. Front. Neurol..

[57] Li C., Li Q., Jiang R., Zhang C., Qi E., Wu M., Zhang M., Zhao H., Zhao F., Zhou H. (2024). Dynamic Changes in Pyroptosis Following Spinal Cord Injury and the Identification of Crucial Molecular Signatures through Machine Learning and Single-Cell Sequencing. J. Pharm. Biomed. Anal..

[58] Jiang D., Yang X., Ge M., Hu H., Xu C., Wen S., Deng H., Mei X. (2023). Zinc Defends against Parthanatos and Promotes Functional Recovery after Spinal Cord Injury through SIRT3-Mediated Anti-Oxidative Stress and Mitophagy. CNS Neurosci. Ther..

[59] Guha L., Singh N., Kumar H. (2023). Different Ways to Die: Cell Death Pathways and Their Association With Spinal Cord Injury. Neurospine.

[60] Becker T., Wullimann M.F., Becker C.G., Bernhardt R.R., Schachner M. (1997). Axonal Regrowth after Spinal Cord Transection in Adult Zebrafish. J. Comp. Neurol..

[61] Crowe M.J., Bresnahan J.C., Shuman S.L., Masters J.N., Beattie M.S. (1997). Apoptosis and Delayed Degeneration after Spinal Cord Injury in Rats and Monkeys. Nat. Med..

[62] Beattie M.S., Farooqui A.A., Bresnahan J.C. (2009). Review of Current Evidence for Apoptosis After Spinal Cord Injury. J. Neurotrauma.

[63] He C., Xu Y., Sun J., Li L., Zhang J.H., Wang Y. (2023). Autophagy and Apoptosis in Acute Brain Injuries: From Mechanism to Treatment. Antioxid. Redox Signal.

[64] Feng H., Wang H., Li J., Ren J., Li Y., Li C., Chen J., Song X., Ning G., Feng S. (2025). Exacerbation of Neuronal Senescence after Spinal Cord Injury: Role of the Macrophage-Derived Transforming Growth Factor-Β1-SMAD2 Signaling Axis. Neural Regen. Res..

[65] Van Niekerk E.A., Tuszynski M.H., Lu P., Dulin J.N. (2015). Molecular and Cellular Mechanisms of Axonal Regeneration After Spinal Cord Injury. Mol. Cell Proteom..

[66] Wu X., Xu X.M. (2016). RhoA/Rho Kinase in Spinal Cord Injury. Neural Regen. Res..

[67] Ma Q., Chen G., Li Y., Guo Z., Zhang X. (2023). The Molecular Genetics of PI3K/PTEN/AKT/MTOR Pathway in the Malformations of Cortical Development. Genes Dis..

[68] Ohtake Y., Hayat U., Li S. (2015). PTEN Inhibition and Axon Regeneration and Neural Repair. Neural Regen. Res..

[69] Guo S., Perets N., Betzer O., Ben-Shaul S., Sheinin A., Michaelevski I., Popovtzer R., Offen D., Levenberg S. (2019). Intranasal Delivery of Mesenchymal Stem Cell Derived Exosomes Loaded with Phosphatase and Tensin Homolog SiRNA Repairs Complete Spinal Cord Injury. ACS Nano.

[70] Mirzaa G.M., Parry D.A., Fry A.E., Giamanco K.A., Schwartzentruber J., Vanstone M., Logan C.V., Roberts N., Johnson C.A., Singh S. (2014). De Novo CCND2 Mutations Leading to Stabilization of Cyclin D2 Cause Megalencephaly-Polymicrogyria-Polydactyly-Hydrocephalus Syndrome. Nat. Genet..

[71] He X., Li Y., Deng B., Lin A., Zhang G., Ma M., Wang Y., Yang Y., Kang X. (2022). The PI3K/AKT Signalling Pathway in Inflammation, Cell Death and Glial Scar Formation after Traumatic Spinal Cord Injury: Mechanisms and Therapeutic Opportunities. Cell Prolif..

[72] Kimura T., Horikoshi Y., Kuriyagawa C., Niiyama Y. (2021). Rho/ROCK Pathway and Noncoding RNAs: Implications in Ischemic Stroke and Spinal Cord Injury. Int. J. Mol. Sci..

[73] Hu J., Selzer M.E. (2017). RhoA as a Target to Promote Neuronal Survival and Axon Regeneration. Neural Regen. Res..

[74] Danilov C.A., Thein T.Z., Tahara S.M., Schönthal A.H., Chen T.C. (2023). Intranasal Delivery of MiR133b in a NEO100-Based Formulation Induces a Healing Response in Spinal Cord-Injured Mice. Cells.

[75] Gwak S.J., Macks C., Jeong D.U., Kindy M., Lynn M., Webb K., Lee J.S. (2017). RhoA Knockdown by Cationic Amphiphilic Copolymer/SiRhoA Polyplexes Enhances Axonal Regeneration in Rat Spinal Cord Injury Model. Biomaterials.

[76] Li Y., Gao Y., Xu X., Shi R., Liu J., Yao W., Ke C. (2017). Slit2/Robo1 Promotes Synaptogenesis and Functional Recovery of Spinal Cord Injury. Neuroreport.

[77] Matamoros A.J., Tom V.J., Wu D., Rao Y., Sharp D.J., Baas P.W. (2019). Knockdown of Fidgetin Improves Regeneration of Injured Axons by a Microtubule-Based Mechanism. J. Neurosci..

[78] Baker L., Tar M., Kramer A.H., Villegas G.A., Charafeddine R.A., Vafaeva O., Nacharaju P., Friedman J., Davies K.P., Sharp D.J. (2021). Fidgetin-like 2 Negatively Regulates Axonal Growth and Can Be Targeted to Promote Functional Nerve Regeneration. JCI Insight.

[79] Smith A.N., Nagrabski S., Baker L., Kramer A.H., Sharp D.J., Byrnes K.R. (2025). Fidgetin-like 2 Knockdown Increases Acute Neuroinflammation and Improves Recovery in a Rat Model of Spinal Cord Injury. J. Neuroinflamm..

[80] Shupp A., Casimiro M.C., Pestell R.G. (2017). Biological Functions of CDK5 and Potential CDK5 Targeted Clinical Treatments. Oncotarget.

[81] Ao C., Li C., Chen J., Tan J., Zeng L. (2022). The Role of Cdk5 in Neurological Disorders. Front. Cell Neurosci..

[82] Zhou F.Q., Zhou J., Dedhar S., Wu Y.H., Snider W.D. (2004). NGF-Induced Axon Growth is Mediated by Localized Inactivation of GSK-3β and Functions of the Microtubule plus End Binding Protein APC. Neuron.

[83] Ahmed Z., Morgan-Warren P.J., Berry M., Scott R.A.H., Logan A. (2019). Effects of SiRNA-Mediated Knockdown of GSK3β on Retinal Ganglion Cell Survival and Neurite/Axon Growth. Cells.

[84] Han Q., Xie Y., Ordaz J.D., Huh A.J., Huang N., Wu W., Liu N., Chamberlain K.A., Sheng Z.H., Xu X.M. (2020). Restoring Cellular Energetics Promotes Axonal Regeneration and Functional Recovery after Spinal Cord Injury. Cell Metab..

[85] Cheng X.T., Huang N., Sheng Z.H. (2022). Programming Axonal Mitochondrial Maintenance and Bioenergetics in Neurodegeneration and Regeneration. Neuron.

[86] Xu X., Li H., Lu S., Shen Y. (2023). Roles of Syntaphilin and Armadillo Repeat-Containing X-Linked Protein 1 in Brain Injury after Experimental Intracerebral Hemorrhage. Neurosci. Lett..

[87] Jin L.-Y., Li J., Wang K.-F., Xia W.-W., Zhu Z.-Q., Wang C.-R., Li X.-F., Liu H.-Y. (2021). Blood-Spinal Cord Barrier in Spinal Cord Injury: A Review. J. Neurotrauma.

[88] Park C.S., Lee J.Y., Seo K.J., Kim I.Y., Ju B.G., Yune T.Y. (2024). TRPM7 Mediates BSCB Disruption After Spinal Cord Injury by Regulating the MTOR/JMJD3 Axis in Rats. Mol. Neurobiol..

[89] Park C.S., Lee J.Y., Choi H.Y., Yune T.Y. (2022). Suppression of Transient Receptor Potential Melastatin 7 by Carvacrol Protects against Injured Spinal Cord by Inhibiting Blood-Spinal Cord Barrier Disruption. J. Neurotrauma.

[90] Xiao X., Li W., Rong D., Xu Z., Zhang Z., Ye H., Xie L., Wu Y., Zhang Y., Wang X. (2021). Human Umbilical Cord Mesenchymal Stem Cells-Derived Extracellular Vesicles Facilitate the Repair of Spinal Cord Injury via the MiR-29b-3p/PTEN/Akt/MTOR Axis. Cell Death Discov..

[91] Li Z., Zhang Z., Zhao L., Li H., Wang S., Shen Y. (2014). Bone Marrow Mesenchymal Stem Cells with Nogo-66 Receptor Gene Silencing for Repair of Spinal Cord Injury. Neural Regen. Res..

[92] Wu J., Zhao Z., Zhu X., Renn C.L., Dorsey S.G., Faden A.I. (2016). Cell Cycle Inhibition Limits Development and Maintenance of Neuropathic Pain Following Spinal Cord Injury. Pain.

[93] Wang Y., Kong Q.J., Sun J.C., Xu X.M., Yang Y., Liu N., Shi J.G. (2018). Protective Effect of Epigenetic Silencing of CyclinD1 against Spinal Cord Injury Using Bone Marrow-Derived Mesenchymal Stem Cells in Rats. J. Cell Physiol..

[94] Baklaushev V.P., Durov O.V., Kalsin V.A., Gulaev E.V., Kim S.V., Gubskiy I.L., Revkova V.A., Samoilova E.M., Melnikov P.A., Karal-Ogly D.D. (2021). Disease Modifying Treatment of Spinal Cord Injury with Directly Reprogrammed Neural Precursor Cells in Non-Human Primates. World J. Stem Cells.

[95] Xiong T., Xiao X., Zhao H., Yang W., Gao X., Yang K., Zheng K., Ji Y., Xu D., Fu R. (2025). The Key to Spinal Cord Recovery: Harnessing P21 Inhibition to Boost Neural Stem/Progenitor Cell Proliferation. ACS Nano.

[96] Zhu S., Diao S., Liu X., Zhang Z., Liu F., Chen W., Lu X., Luo H., Cheng X., Liao Q. (2025). Biomaterial-Based Strategies: A New Era in Spinal Cord Injury Treatment. Neural Regen. Res..

[97] Gao Y., Wang K., Wu S., Wu J., Zhang J., Li J., Lei S., Duan X., Men K. (2024). Injectable and Photocurable Gene Scaffold Facilitates Efficient Repair of Spinal Cord Injury. ACS Appl. Mater. Interfaces.

[98] Dong X., Lu Y., Hu Q., Zeng C., Zheng J., Huang J., Dong H., Zou P., Wang T., Wu Y. (2025). Engineered Exosome-Loaded Silk Fibroin Composite Hydrogels Promote Tissue Repair in Spinal Cord Injury Via Immune Checkpoint Blockade. Small.

[99] Cheriyan T., Ryan D.J., Weinreb J.H., Cheriyan J., Paul J.C., Lafage V., Kirsch T., Errico T.J. (2014). Spinal Cord Injury Models: A Review. Spinal Cord.

[100] Sobolev V.E., Sysoev Y.I., Vyunova T.V., Musienko P.E. (2025). Animal Models of Spinal Cord Injury. Biomedicines.

[101] Chen X., Mangala L.S., Rodriguez-Aguayo C., Kong X., Lopez-Berestein G., Sood A.K. (2018). RNA Interference-Based Therapy and Its Delivery Systems. Cancer Metastasis Rev..

[102] Tatiparti K., Sau S., Kashaw S., Iyer A. (2017). SiRNA Delivery Strategies: A Comprehensive Review of Recent Developments. Nanomaterials.

[103] Tang Q., Khvorova A. (2024). RNAi-Based Drug Design: Considerations and Future Directions. Nat. Rev. Drug Discov..

[104] Kurreck J. (2005). RNA Interference: Perspectives and Caveats. J. RNAi Gene Silenc..

[105] Meng Z., Lu M. (2017). RNA Interference-Induced Innate Immunity, Off-Target Effect, or Immune Adjuvant?. Front. Immunol..

[106] Xiao H., Amarsaikhan O., Zhao Y., Yu X., Hu X., Han S., Chaolumen Baigude H. (2023). Astrocyte-Targeted SiRNA Delivery by Adenosine-Functionalized LNP in Mouse TBI Model. Mol. Ther. Nucleic Acids.

[107] Zhou Z., Tian X., Tian X., Mo B., Xu H., Zhang L., Huang L., Yao S., Huang Z., Wang Y. (2020). Adipose Mesenchymal Stem Cell Transplantation Alleviates Spinal Cord Injury-Induced Neuroinflammation Partly by Suppressing the Jagged1/Notch Pathway. Stem Cell Res. Ther..

[108] Powell J.E., Lim C.K.W., Krishnan R., McCallister T.X., Saporito-Magriña C., Zeballos M.A., McPheron G.D., Gaj T. (2022). Targeted Gene Silencing in the Nervous System with CRISPR-Cas13. Sci. Adv..

[109] Zhang H., Li D., Zhang Y., Li J., Ma S., Zhang J., Xiong Y., Wang W., Li N., Xia L. (2018). Knockdown of LncRNA BDNF-AS Suppresses Neuronal Cell Apoptosis via Downregulating MiR-130b-5p Target Gene PRDM5 in Acute Spinal Cord Injury. RNA Biol..

[110] Zhang X.-M., Zeng L.-N., Yang W.-Y., Ding L., Chen K.-Z., Fu W.-J., Zeng S.-Q., Liang Y.-R., Chen G.-H., Wu H.-F. (2022). Inhibition of LncRNA Vof-16 Expression Promotes Nerve Regeneration and Functional Recovery after Spinal Cord Injury. Neural Regen. Res..

[111] Cui S.-Y., Zhang W., Cui Z.-M., Yi H., Xu D.-W., Liu W., Zhu X.-H. (2021). Knockdown of Long Non-Coding RNA LEF1-AS1 Attenuates Apoptosis and Inflammatory Injury of Microglia Cells Following Spinal Cord Injury. J. Orthop. Surg. Res..

[112] Chen P., Huang Z., Liu Y. (2025). LncRNA ZFAS1 Combined with SRSF1 Regulate CNPY2 Expression and Leads to Microglia Endoplasmic Reticulum Stress–Induced Spinal Cord Injury. Mol. Neurobiol..

