# Supplementary material for: RNA Interference and Its Key Targets for Spinal Cord Injury Therapy: What Is Known So Far?
Source: Int J Mol Sci. 2025 Oct 10;26(20):9861. doi: 10.3390/ijms26209861 (PMC12563420; doi:10.3390/ijms26209861)
Supplement: Supplementary file 1 [file ijms-26-09861-s001.zip › ijms-3857657-supplementary.pdf]

Target	SCI model / species (models of other injuries, cell models)	Timing of application	Delivery method/ RNAi tool	Treatment outcome	Reference
RGMa	Compression SCI / rats	1 h after surgery, 3 mm next to lesion site. Injection repeated twice a week for 6 weeks	shRNA, lentiviral vector	Improved BBB scores from week 2–3, increased ladder success rate with fewer slips; increased NF-200 and synaptophysin in gray matter	[5]
IKKβ/NF-κB	Compression SCI / rats	24 hours after SCI, miR-199b was injected once a week for six weeks.	synthetic miR-199b mimic; intraspinal/vertebral canal injection	Reduced IKKβ-NF-κB signaling, reduced expression of TNF-α and IL-1β, increased grip strength and motor activity	[6]
ChPF	Glial cell cultures from newborn rats (1 day postnatal)<br><br>Neu7 cell line	Astrocytes ~7-10 days<br><br>Neu7 up to 3-4 days	pSuppressorNeo vector (IMG-800) encoding ChPF shRNA<br>Neu7 cells were transfected using the Amaxa nucleofection technology	Reduced CSPG expression, enhanced neurite outgrowth of cerebellar granule neurons in culture, enhanced axon growth (maximal effect reached on day 2-3)	[19]
Tenascin-C	Transient middle cerebral artery occlusion (ischemic stroke) / mice	4 h after Middle Cerebral Artery Occlusion via intracerebroventricular injection	Injection via a retroorbital route with Ambion VR Silencer™ Select in vivo grade TNC siRNA (or negative control siRNA) mixed with polyethylene glycol-liposome	Significantly reduced infarct volume (day 3 of reperfusion), improved post-stroke motor function recovery	[21]
EphrinB3	Contusion SCI / rats	Immediately after injury	Intrathecal delivery of lentiviral pGCSIL-RNAi-GFP vectors encoding	Increased motor activity, increased numbers of GAP-43+ axonal fibers	[27]

			ephrinB3 siRNA (LV-siRNA1/2)		
Semaphorin 3A	Compression SCI / rats	Immediately after injury	Sema 3A siRNA–gold nanoparticle complexes (AuNP@siRNA-Sema3A)	Increase of motor activity,<br>significant increase in cell survival and neuronal differentiation of the transplanted NSCs after SCI	[28]
CTGF	Contusion SCI / rats	Immediately after SCI, then repeatedly for 5 consecutive days beginning on day of injury.	Exosomes from rat bone marrow-derived MSCs, loaded with synthetic CTGF siRNA were administered via tail vein injection	Significant reduction of CTGF and glial scar proteins (GFAP, vimentin, fibronectin, laminin), decreased astrogliosis, reduced inflammation and neuronal apoptosis, upregulation of BDNF and TGF-β1, improved locomotor and urinary function, enhanced axon regeneration, increased BBB and neurophysiological recovery scores	[30]
PLK4	Contusion SCI / rats	Immediately after the injury	siRNA targeting PLK4 encapsulated in PLGA nanoparticles, injected locally into the parenchyma around the injury site	Increase of motor activity.<br>promoted motor functional recovery,<br>decrease of astrocyte proliferation and inflammatory responses	[31]
Lipocalin 2, GFAP and Vimentin	Contusion SCI /mice	Immediately post-injury, delivered directly into the lesion	Intralesional injection of siRNA-3WJ nanoparticles	Reduced astroglial protein secretion, limiting inflammation in quiescent astrocytes.	[32]
Lipocalin 2	Contusion SCI / mice	Anti-Lcn2 3WJ, injected immediately after SCI into the lesion epicenter; iNSCs at 7 days post-injury.	Injection of packaging RNA (pRNA) 3-way-junction nanostructures carrying anti-Lcn2 siRNA (3WJ-L12)	Increase of motor activity<br>Reduced glial scar volume Increased neuronal survival	[34]
OASIS	Contusion SCI	5 days after SCI	anti-OASIS siRNA or	Attenuated astrogliosis, but impaired	[35]

(CREB3L1)	/ mice		scrambled siRNA mixed with PTD-DRBD were injected into the center and periphery of the injured lesion	motor recovery after SCI	
SASH1	Contusion SCI / rats	Immediately after injury	Small interfering RNA against SASH1 (siSASH1)	SASH1 knockdown reduced GFAP expression in vivo, Improved BBB locomotor scores after SCI; increased axonal growth when neurons cocultured with SASH1-knockdown astrocytes	[36]
Ephrin-B2	Astroglial-fibrotic scar-like cell clusters	siRNA was applied concurrently with TGF-β1 at the initiation of scar formation in vitro	Direct addition of synthetic ephrin-B2 siRNA into the coculture medium (microfluidic platform) to induce RNAi in astrocytes/ meningeal fibroblasts.	Significantly reduced both the number and the diameter of cell clusters induced by TGF-β1, diminished expression of aggrecan and versican	[38]

IRF5	Contusion SCI / mice	1 hour after SCI (single IV injection), follow-up up to 28–42 days	Intravenous injection of lipidoid nanoparticle-encapsulated IRF5 siRNA via tail vein	Selective knockdown of IRF5 in lesional macrophages led to strong M1→M2 shift, inflammation resolution, better white matter/myelin/neurofilament preservation, and durable motor recovery approach exploits immune cell uptake of nanoparticles	[42]
IL-1β	Contusion SCI / rats	Injection 48h before contusion	Lentiviral siRNA vector, intraspinal injection	Increase of motor activity<br>Increased AKT1 expression	[43]
HuR	Contusion SCI / mice	In vitro, 72 h before stretch injury	Astrocytes were electroporated with siRNA specific to HuR (siHuR) using the Neon transfection kit	Reduced cytokine and chemokine induction, attenuated chemoattraction of neutrophils/microglia by astrocytes	[44]

MIF + GDNF	Transection SCI / mice	Immediately after SCI	Injectable photocurable lipid nanoparticle GelMA (PLNG) scaffold loaded with siRNA against MIF (siMIF) and GDNF protein, locally administered and in situ photocured	Combined local delivery of MIF siRNA and GDNF with a biodegradable injectable scaffold achieves synergistic anti-inflammatory and neuroregenerative repair after SCI in mice.	[45]
TMEM173 / STING	Compression SCI / mice, including Sting-knockout and transgenic (KO/TG) models	-	The study used KO/TG mice for in vivo analysis and adenoviral shRNA for in vitro knockdown	Sting KO mice showed alleviated inflammatory response and improved locomotor recovery after SCI	[51]
RhoA	Contusion SCI / mice	Initiated 24h post-injury; injected daily for 3	Tail vein co-injection of miR133b mimic with	Intravenous miR133b/Ago2 accumulates specifically in	[74]

		consecutive days	Argonaute-2 protein (3 days)	injured cord, downregulates ECM and inflammation at lesion, reduces D/V scar size, no apparent off-target effect or toxicity.	
RhoA	Compression SCI / rats	Immediately after injury	PgP/siRhoA polyplexes (nanoparticle of poly(lactide-co-glycolide)-g-polyethylenimine with siRNA) injection into SCI lesion.	Reduced neuronal apoptosis and glial scarring, and enhances axonal regeneration. Note: repeated dosing superior to single for anatomical outcomes	[75]
Robo1	Contusion SCI / rats	Immediately after injury	RNAi lentivirus injected into the parenchyma near the contusive site	Decrease in RhoA protein levels, promoted functional recovery after SCI	[76]
FL2	Cavernous nerve injury (peripheral nerve injury model) / rats	Immediately after injury, and post-injury treatments (acute phase)	Local application of FL2-siRNA encapsulated in nanoparticles or microgel wafers at injury site	Promoted significant axonal regeneration, enhanced nerve regrowth across transection gaps, reduced growth cone collapse	[78]
FL2	Contusion SCI / rats	Immediately after injury	Nanoparticle-encapsulated siRNA delivered intrathecally at lesion epicenter (T9)	Increased acute pro- and anti-inflammatory immune responses, promoted microglial accumulation early, then reduced chronic inflammation Note: FL2 expression is acutely upregulated after SCI particularly at lesion site and rostral sections.	[79]
SNPH	Cervical C5 dorsal hemisection, unilateral	SCI induced in the beginning of the study	Gene deletion	Deletion of Snph restored mitochondrial membrane potential after injury by removing damaged mitochondria and replenishing healthy ones; promoted axon regeneration,	[84]

	pyramidotomy, and thoracic T8 complete transection / mice, including Sting-knockout and transgenic models			synapse formation, and functional recovery after SCI	
SNPH	ICH (intracerebral hemorrhage) via autologous blood injection / mice	Stereotactic AAV9-hSyn-SNPH shRNA injection, 3 weeks prior to ICH	Stereotaxic AAV9-hSyn-SNPH shRNA (neurons, perihematomal)	Reduced neuronal cell death, improved mitochondrial axon distribution/localization, and significantly improved neurobehavioral and sensorimotor outcomes (neurological score, foot fault, adhesive removal test)	[86]
PTEN	Compression SCI / rats	At 24 hours after the operation	Intravenous administration of HucMSC-derived extracellular vesicles (EVs) with miR-29b-3p Note: miR-29b-3p targets PTEN	Improved motor function, reduced pathological changes, promoted nerve function repair. Inhibition of miR-29b-3p or overexpression of PTEN reversed beneficial effect of EVs	[90]
NgR	Transaction SCI / rats	6 hours after the injury	siRNA delivered to BMSCs via Lipofectamine 2000, then transplantation of cells via tail vein	Silencing Nogo-66 receptor in BMSCs before transplantation greatly enhances structural and functional recovery after SCI compared to BMSCs only or no treatment groups.	[91]
Cyclin D1	Hemisection injury / rats	6 hours after the injury	BMSCs transfected with Cyclin D1 siRNA plasmid, stereotaxic local transplantation into injured spinal cord	Silencing Cyclin D1 in BMSCs enhances their therapeutic effects post-SCI, leading to better locomotor function and tissue regeneration compared to treatment with just BMSCs or saline; likely mechanisms of action: reduced inflammation, enhanced astrocyte/neuron survival	[93]

PTEN / MIF	Transaction SCI / mice	Local scaffold injection immediately after transection; three doses total, once weekly; scaffold degrades and releases siRNA over 7 days per dose	PLNG GelMA hydrogel loaded with both siPTEN and siMIF LNP–siRNA complexes; blue light photocuring	siPTEN promoted neuron axon regeneration and branching; dual siPTEN+siMIF outperformed single-target groups in BMS and histology; growth factor upregulation (EGF, bFGF, IGF-I) and pathway activation (PI3K-Akt/MAPK/JAK-STAT) with scaffold treatment	[97]
Jagged1	Contusion SCI / mice	GFP-labeled ADSCs were injected into the lesion site immediately after the injury Jagged1 siRNA was injected into the spinal cord 72 h before SCI	GFP-labeled ADSCs were injected directly into the SCI epicenter. A lentiviral vector encoding Jagged1 siRNA was injected intra-parenchyally into the SC, 3 days before SCI.	Increased motor function and activity, decreased levels of SCI-induced proinflammatory cytokines and microglial activation, increased neuronal survival, lower expression of proinflammatory mediator when combined with stem cell treatment	[107]
BDNF-AS	Compression SCI / rats	-	Intrathecal injection of lentiviral siRNA-BDNF-AS (LV-siRNA-BDNF-AS)	Alleviates apoptosis, and promotes functional recovery	[109]
LEF1-AS1	Contusion SCI / rats	-	shRNA/siRNA-mediated knockdown of LEF1-AS1 (sh-LEF1-AS1)	LEF1-AS1 is upregulated after SC, its knockdown confers protection by increasing miR-222-5p and suppressing RAMP3, thereby attenuating microglial apoptosis and inflammation	[110]
Vof-16	Transaction SCI / rats	injection into lesion area immediately post-injury	Lentiviral knockdown (pHBLV-U6-MCS-CMV-ZsGreen-PGK-PURO)	Early inhibition of Vof-16 enhances axonal regeneration and functional recovery; knockdown elevates Bcl-2 and reduces TNF-α/Caspase-3 in vivo; findings suggest Vof-16 modulates inflammation and apoptosis after SCI	[110]
